# Epidemiology of protozoan and helminthic parasites in wild passerine birds of Britain and Ireland

**DOI:** 10.1017/S0031182022001779

**Published:** 2023-03

**Authors:** Fatemeh (Rose) Parsa, Sam Bayley, Fraser Bell, Stephen Dodd, Ray Morris, Jean Roberts, Denise Wawman, Simon R. Clegg, Jenny C. Dunn

**Affiliations:** 1 Department of Life Sciences, University of Lincoln, Joseph Banks Laboratories, Lincoln, Lincolnshire LN6 7TS, UK; 2 Siskin, Keelnacronagh West, Enniskeane, Cork P47 NP90, Ireland; 3 Centre for Ecology and Conservation, University of Exeter, Cornwall Campus, Penryn, Cornwall TR10 9FE, UK; 4 RSPB Centre for Conservation Science, The Lodge, Sandy, Bedfordshire SG19 2DL, UK; 5 RSPB Centre for Conservation Science, Llys Castan, Parc Menai, Bangor, Wales LL57 4FH, UK; 6 Marden Wildlife, Dirt House, Summerhill Road, Marden, Kent TN12 9DB, UK; 7 British Trust for Ornithology, Thetford IP24 2PU, UK; 8 Department of Zoology, Edward Grey Institute of Field Ornithology, Oxford University, Oxford, UK

**Keywords:** Abundance, birds, British Isles, epidemiology, infection intensity, intestinal parasites, *Isospora*, prevalence, *Syngamus*

## Abstract

Avian endoparasites play important roles in conservation, biodiversity and host evolution. Currently, little is known about the epidemiology of intestinal helminths and protozoans infecting wild birds of Britain and Ireland. This study aimed to determine the rates of parasite prevalence, abundance and infection intensity in wild passerines. Fecal samples (*n* = 755) from 18 bird families were collected from 13 sites across England, Wales and Ireland from March 2020 to June 2021. A conventional sodium nitrate flotation method allowed morphological identification and abundance estimation of eggs/oocysts. Associations with host family and age were examined alongside spatiotemporal and ecological factors using Bayesian phylogenetically controlled models. Parasites were detected in 20.0% of samples, with corvids and finches having the highest prevalences and intensities, respectively. *Syngamus* (33%) and *Isospora* (32%) were the most prevalent genera observed. Parasite prevalence and abundance differed amongst avian families and seasons, while infection intensity varied between families and regions. Prevalence was affected by diet diversity, while abundance differed by host age and habitat diversity. Infection intensity was higher in birds using a wider range of habitats, and doubled in areas with feeders present. The elucidation of these patterns will increase the understanding of parasite fauna in British and Irish birds.

## Graphical abstract

**Figure:**
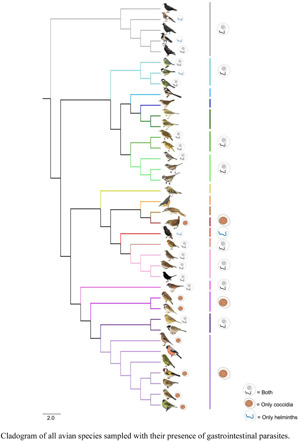

## Introduction

Avian endoparasites play important roles in biodiversity, behaviour, ecology, host evolution and species conservation (Loye and Carroll, 1995; Richner, 1998; Asakawa *et al*., 2002; Hudson *et al*., 2006). It is therefore important to understand host–parasite associations in order to predict patterns of parasite emergence, transmission and pathogenicity (Poulin *et al*., 2011). The pervasiveness and wide variety of parasites capable of infecting a host can have distinct implications for both inter- and intraspecific transmissions. More infected individuals can increase the probability of disease transmission in a community, underlining the importance of understanding the factors that influence parasite prevalence (Pérez-Tris and Bensch, 2005).

The intricate life cycles of certain helminths, such as *Fasciola* spp. have co-evolved with their hosts' complex food-webs due to their requirements of definitive, paratenic and intermediate hosts (Hoberg, 1996; Bakker *et al*., 1997). As birds are globally widespread in nature and can act as reservoirs for various generalist parasites, they may play an important role in cross-species transmission (Ishtiaq and Renner, 2020); their high sociality can also have implications for disease transmission (Brown and Brown, 2004). Identifying the trends that occur in parasite emergence is also significant for the conservation of non-adapted species. Immunologically naïve animals are often more sensitive to infection due to the lack of coevolution with the introduced parasite, which may lead to the development of severe symptoms and mortality (Howe *et al*., 2012; Vanstreels *et al*., 2014). Therefore, determining the patterns of association of parasitic infections in wild bird populations is indispensable.

There is a dearth of epidemiological studies on the occurrence and distribution of intestinal parasites infecting wild passerine populations; elucidating these host–parasite associations is essential in understanding transmission, emergence and virulence (Waldenström *et al*., 2002; Pérez-Tris and Bensch, 2005; Penczykowski *et al*., 2016). Prior epidemiological research has often revolved around livestock, pets, humans and other mammalian wildlife (Hoque *et al*., 2014; Minetti *et al*., 2014), but only a few studies have examined both helminths and protozoa in wild birds (e.g. Wascher *et al*., 2012). The only study conducted in Britain to include wild passerines solely examined coccidia (Brown *et al*., 2010). There have been other studies examining the intestinal parasites of birds, including some in Asia and South America, that use a wide range of detection techniques such as staining, sedimentation and sporulation (Badparva *et al*., 2014; Hoque *et al*., 2014); however, the majority examined domestic birds or those kept in zoological gardens (Pérez Cordón *et al*., 2009). Therefore, observational epidemiological studies are integral in identifying the shared parasite fauna of wild birds.

Moreover, there is a scarcity of research on the potential effects of ecological variables, such as anthropogenic food availability, habitat and diet, on the prevalence, abundance and infection intensity of passerine intestinal parasites (but see Bandelj *et al*., 2015). The effect of humans providing supplementary feeding to wildlife, such as through the use of supplementary bird seed, has been linked to the spread of *Trichomonas gallinae* protozoa, the aetiological agent of avian trichomonosis, to naïve hosts (e.g. Lennon *et al*., 2013). Meanwhile, the diversity of the habitats and diets shared by passerines can impact their exposure to, or resistance against, parasites (Becker *et al*., 2018; Strandin *et al*., 2018); although these can be intertwined with other factors, such as migration, nutrition and feeding behaviour, they are still necessary to examine to elucidate whether they explain any trends observed in the rates of infections.

Coinfection, or the state of being infected by multiple pathogens simultaneously, is ubiquitous in nature, with various types of endoparasites often being detected together in wild vertebrates (Ezenwa, 2016). Coinfections can affect host health alongside influencing parasite transmission (Cox, 2001). A meta-analysis by Knowles (2011) demonstrated that helminths can influence immune system modulation of the murine (*Mus musculus*) host, leading to differing impacts on malaria infection dynamics. Despite the important dynamic effects multiple parasites can have on each other and the host (Thomas *et al*., 2022), there is a scarcity of research into avian parasite epidemiology while taking coinfection into account.

Here, we determine the prevalence and abundance of helminths and coccidians infecting a range of wild avian hosts at 13 geographically separate sites across Britain and Ireland. We test whether parasite prevalence, abundance and infection intensity differ between avian host families, ages and spatiotemporally, and determine whether parasite genus influences the trends in parasite abundance and intensity; we also test the impacts of parasite genus on these variables separately. We also test whether the presence of feeders could act as a potential source of parasite transmission, predicting they will be linked with increased parasite prevalence and abundance. We also determine whether greater host habitat and diet diversity will increase both prevalence and abundance of gut parasites across avian hosts due to heightened exposure. Finally, we carry out rarefaction analyses to examine expected parasite diversity within each host species and test for under-sampling, and examine patterns of coinfection to test whether some commonly occurring parasites are detected together more or less often than would be expected.

## Methods

### Study sites

**Fig. 1.**
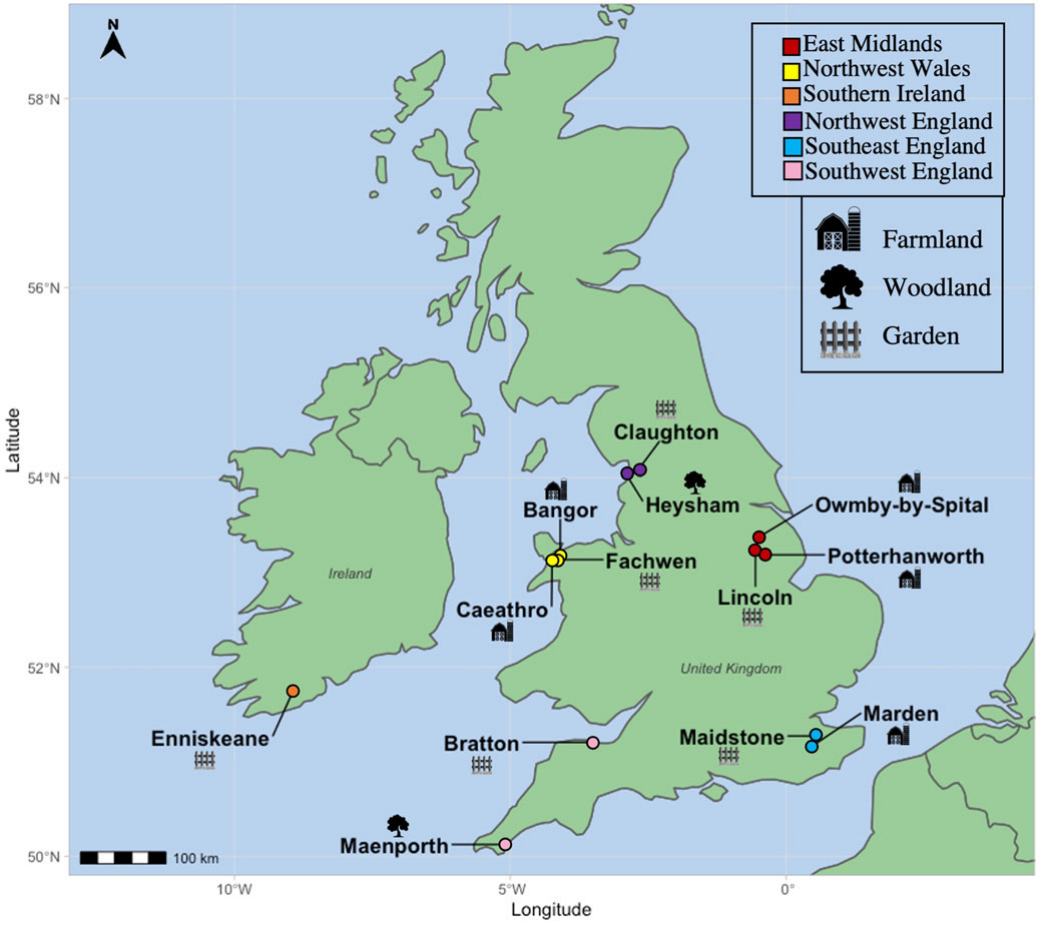
Locations of avian fecal sampling sites throughout the British Isles, with the legends showing their characterization into geographical regions as well as symbols denoting the primary habitat type found at these areas.

Fecal samples were collected from birds caught as part of regular ringing operations over 15 months, from March 2020 to June 2021 at 13 sites in England (*n* = 9), Wales (*n* = 3) and the Republic of Ireland (*n* = 1). Sites consisted of garden (*n* = 6), woodland (*n* = 2) or farmland (*n* = 5) habitats across 7 counties (Fig. 1).

### Sample collection

Birds were caught using whoosh or mist nests on days with minimal wind or rain (time range: 6 am–4:30 pm; median: 9:47 am), fitted with a British Trust for Ornithology metal ring, and aged and sexed, where possible, according to standard techniques (Svensson, 1992); only 1 bird, a blue tit, appeared in slightly poor condition with some facial feather loss. The presence or absence of feeders at each ringing site was also recorded. Birds were retained for a maximum of 10 min in sterile holding bags to allow for the production of a fecal sample prior to release. Feces were placed into clean, pre-labelled 1.5 mL tubes and refrigerated within 8 h in plastic bags until postage; the specimens were posted as soon as feasible for laboratory analysis (range: 2–14 days; median: 5 days).

### Laboratory analysis

A conventional fecal flotation technique allowed for the detection of helminth eggs and protozoan oocysts using sodium nitrate flotation fluid with a specific gravity of 1.2 (Vetlab Supplies Ltd, Pulborough, West Sussex, UK) and the McMaster egg counting technique (Levecke *et al*., 2011); the weights of the feces were measured (Supplementary Table 2) and scaled up to calculate the eggs/oocysts per g (EPG/OPG) to standardize the quantification of parasite abundance and intensity. Published keys assisted in morphological identification to the genus level based on colour, shape, size and other distinguishable features (Svobodová, 1994; Presswell and Lagrue, 2016; Gallo *et al*., 2018).

### Phylogenetically controlled mixed effects models

Statistical analyses were performed in R version 4.1.0 (R Core Team, 2021). Three phylogenetically controlled generalised linear mixed-effects models (PGLMMs) were constructed using the *MCMCglmm* package (Hadfield, 2010) for Bayesian analysis, alongside the *ape* package (Paradis *et al*., 2004) to identify spatiotemporal and ecological associations with parasite prevalence, abundance and infection intensity when controlling for host phylogeny and time until analysis. The Markov chain Monte Carlo (MCMC) allows the analysis of complex models using a probabilistic sampling technique while controlling for fine-scale phylogenetic relatedness by treating avian phylogeny as a random variable. The phylogeny was obtained from BirdTree (Jetz *et al*., 2012) using the Ericson All Species model. Due to systematic reclassifications of 2 former subspecies to distinct taxa, no data were available on BirdTree for *Corvus cornix* and *Acanthis cabaret*, so data for *Corvus corone* and *Carduelis flammea* were used as replacements due to being the most closely related species.

The response variable for the parasite prevalence model comprised of the absence or presence (0 or 1) of any parasite genus within each host; this model comprised of a binomial PGLMM with a logit link. Parasite abundance and infection intensity were defined as the number of eggs/oocysts combined within each individual or each infected individual, respectively (Reiczigel *et al*., 2019); these were each analysed using Poisson-distributed PGLMMs with a log link. Fixed effects in all 3 models comprised of bird family, host age (adult or juvenile/first year), season (a 4-level categorical variable: winter: December–February, *n* = 157; spring: March–May, *n* = 196; summer: June–August, *n* = 127; autumn: September–November, *n* = 189), geographic region [a 6-level categorical variable: region with 4 areas in England, 1 in Wales and 1 in Ireland (Fig. 1)], bird feeder presence or absence and diet and habitat diversity at the species level; these latter continuous variables were extracted from a Europe-wide dataset (Storchová and Hořák, 2018) and denote the total number of diets or habitats known to be used by the species (diet range: 1–6; habitat range: 1–5). Only families with samples from more than 10 individuals were included in the analyses, so Cettiidae (*n* = 1), Certhiidae (*n* = 2), Sittidae (*n* = 2) and Regulidae (*n* = 5) were removed from the dataset. To include host age in the analyses, specimens from *Aegithalos caudatus* (*n* = 13), *Passer montanus* (*n* = 6) and *Passer domesticus* (*n* = 15) were removed from the analysis as they undergo a complete post-juvenile moult and cannot be aged during ringing activities after the autumn; birds whose ages were not recorded (*n* = 42) were also removed from the analysis, resulting in final sample sizes of 669 (prevalence, abundance) and 138 (infection intensity) birds. For the abundance and infection intensity models, the analyses excluded Phylloscopidae (*n* = 18) as they were uninfected. The abundance and infection intensity analyses were also repeated to include parasite genus in the model to determine whether infection type influences parasite load. Host phylogeny and average time lapsed from sample collection until analysis (in days; range: 2.2–12.8 days) were included as a random effect in all models. Models were run for 500 000 iterations, with a 1000 burn-in period and a 500-thinning interval; the resulting effective sample sizes were similar amongst factors (~998).

**Table 1.** Significant results of phylogenetically controlled, Poisson MCMC GLMMs comparing the various factors affecting avian parasite abundance

		Estimate	95% CI		pMCMC
			Lower	Upper	
Intercept		−5.89	−68.21	60.26	0.812
Avian family					
Sturnidae	Troglodytidae	−25.69	−48.97	−2.46	0.014
Season					
Winter	Autumn	4.01	0.09	6.98	0.012
	Spring	7.94	4.08	11.99	<0.001
Ecological variables					
	Habitat diversity	0.24	−2.37	−2.08	0.024
Host variable					
	Age (juvenile *vs* adult)	2.53	0.36	5.06	0.032
Random effects					
	Host phylogeny	158.20	0.0023	793.70	
	Time until analysis	16 664	0.0030	4876	

Estimates, lower and upper 95% confidence interval (CI) values, and *P* values particle Markov chain Monte Carlo (pMCMC) have been included for the intercept, random effects and each level of the factors.

### Rarefaction analyses

To estimate how many parasites may have been missed due to under-sampling of hosts, rarefaction analyses were conducted using the *vegan* package (Oksanen *et al*., 2017), based on sampling without replacement. This allowed for quantification of any potential sampling biases by estimating the total parasite genera richness that was likely to be detected amongst the various avian host families based on observed samples (Gotelli and Colwell, 2001). Only families with more than 1 parasite genus observed (*n* = 9) were included. If asymptotic richness is shown to be reached by a host, then it can be concluded that the majority of parasite genera present within that host are likely to have been detected.

### Co-occurrence analyses

The *cooccur* package (Griffith *et al*., 2016) was used to test for patterns in parasite co-occurrence, and analyse whether multiple parasite genera were observed together at random or whether there were positive or negative correlations. This was performed by using the overall prevalence of each parasite and examining whether the observed rate of genus cooccurrence was higher or lower than expected (Griffith *et al*., 2016). Trematodes were omitted from this analysis because they were underrepresented in the data and to meet the assumption that only parasites present in more than 5% of positive samples are included.

## Results

Samples were collected from 18 families of wild Passeriformes (*n* = 755), comprising 28 genera and 38 species. Sample sizes for each species range from 1 to 140 (mean ± s.e.: 19.9 ± 4.2), with sample sizes per family varying from 1 to 228 (41.9 ± 13.2).

**Table 2.** Significant results of phylogenetically controlled, binomial MCMC GLMMs testing whether host and ecological factors influence avian parasite prevalence

		Estimate	95% CI		pMCMC
			Lower	Upper	
Intercept		−161.10	−1088.33	911.47	0.675
Avian family					
Acrocephalidae *vs*	Corvidae	767.12	0.58	1549.19	0.032
	Phylloscopidae	−2604.02	−6098.42	−80.49	0.018
Corvidae *vs*	Emberizidae	−694.84	−1221.29	−59.10	0.030
	Fringillidae	−767.33	−1266.14	−11.35	0.018
	Muscicapidae	−541.16	−1001.43	−37.98	0.034
	Paridae	−819.39	−1292.95	−149.24	0.016
	Phylloscopidae	−1456.88	−2471.92	−460.68	0.004
	Sylviidae	−718.54	−1219.41	−71.39	0.014
	Troglodytidae	−948.72	−1629.59	−173.59	0.006
Emberizidae *vs*	Phylloscopidae	−81.23	−163.16	−0.11	0.040
Fringillidae *vs*	Phylloscopidae	−381.50	−768.88	−74.62	0.004
	Prunellidae	105.74	0.13	228.02	0.028
	Sturnidae	167.36	9.79	353.28	0.030
	Turdidae	102.49	0.62	231.87	0.046
Paridae *vs*	Sturnidae	597.16	77.44	1061.17	0.024
Phylloscopidae *vs*	Prunellidae	−3271.88	−5012.14	−297.73	0.004
	Sturnidae	−1728.78	−3915.58	−267.10	0.008
	Turdidae	−336.47	−653.96	−5.81	0.016
Sturnidae *vs*	Troglodytidae	−966.31	−1725.63	−179.77	0.012
Troglodytidae *vs*	Turdidae	622.41	100.93	1120.12	0.006
Season					
Autumn *vs*	Spring	−145.35	−277.39	−8.82	0.026
	Winter	114.11	7.51	217.01	0.022
Spring *vs*	Winter	209.83	60.98	333.98	<0.001
Ecological variables					
	Diet diversity	−64.72	−181.32	−41.36	0.023
Host variable					
	Age (juvenile *vs* adult)	91.71	3.28	174.78	0.014
Random effects					
	Host phylogeny	42 829	0.003263	277 474	
	Time until analysis	5 399 675	0.002661	219 631	

Estimates, lower and upper 95% CI values, and *P* values (pMCMC) have been included for the intercept, random effects and each level of the factors.

### Parasite genera

**Fig. 2.**
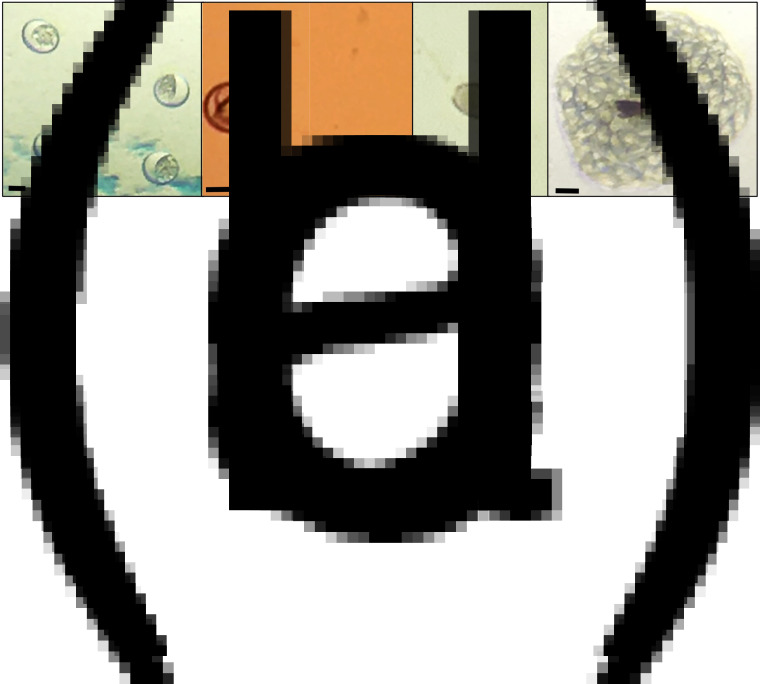
Protozoa: (a) unsporulated and (b) sporulated *Isospora* sp. oocysts (~25 × 23 *μ*m^2^); (c) unsporulated *Eimeria* sp. oocyst (~25 × 15 *μ*m^2^) and (d) *Monocystis* sp. gametocyst (~185 × 170 *μ*m^2^) with enclosed sporozoites. Magnification is 10× (a, c, d) and 20× (b). Scale bar: 10 *μ*m.

**Fig. 3.**
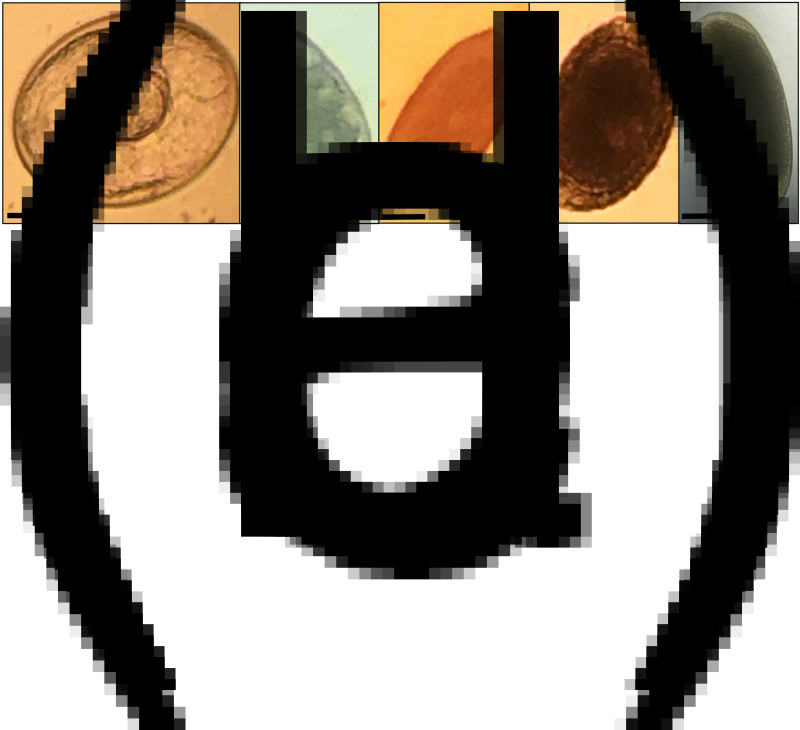
Helminths: cyst of a cestode (a; ~62 × 40 *μ*m^2^); eggs of *Syngamus* sp. (b; ~80 × 45 *μ*m^2^), *Capillaria* sp. (c; ~40 × 23 *μ*m^2^), *Porrocaecum* sp. (d; ~97 × 59 *μ*m^2^) and a trematode (e; ~53 × 25 *μ*m^2^). Magnification is 10× (b, e) and 20× (a, c, d). Scale bar: 10 *μ*m.

Fourteen parasite genera were identified from microscopic analysis of fecal samples following fecal flotation. These comprised of gregarines (*Monocystis*), coccidians (*Eimeria*, *Isospora*), nematodes (*Capillaria*, *Porrocaecum*, *Syngamus*), cestodes (*Anonchotaenia*, *Dilepis*, *Passerilepis*, *Variolepis*) and trematodes (*Brachydistomum*, *Collyriclum*, *Echinostoma*, *Leucochloridium*) (Figs 2 and 3; Supplementary Fig. 1). Although mites and potential larvae-like stages were also detected in the samples, they were not included in fecal egg/oocyst counts.

### Parasite prevalence

**Fig. 4.**
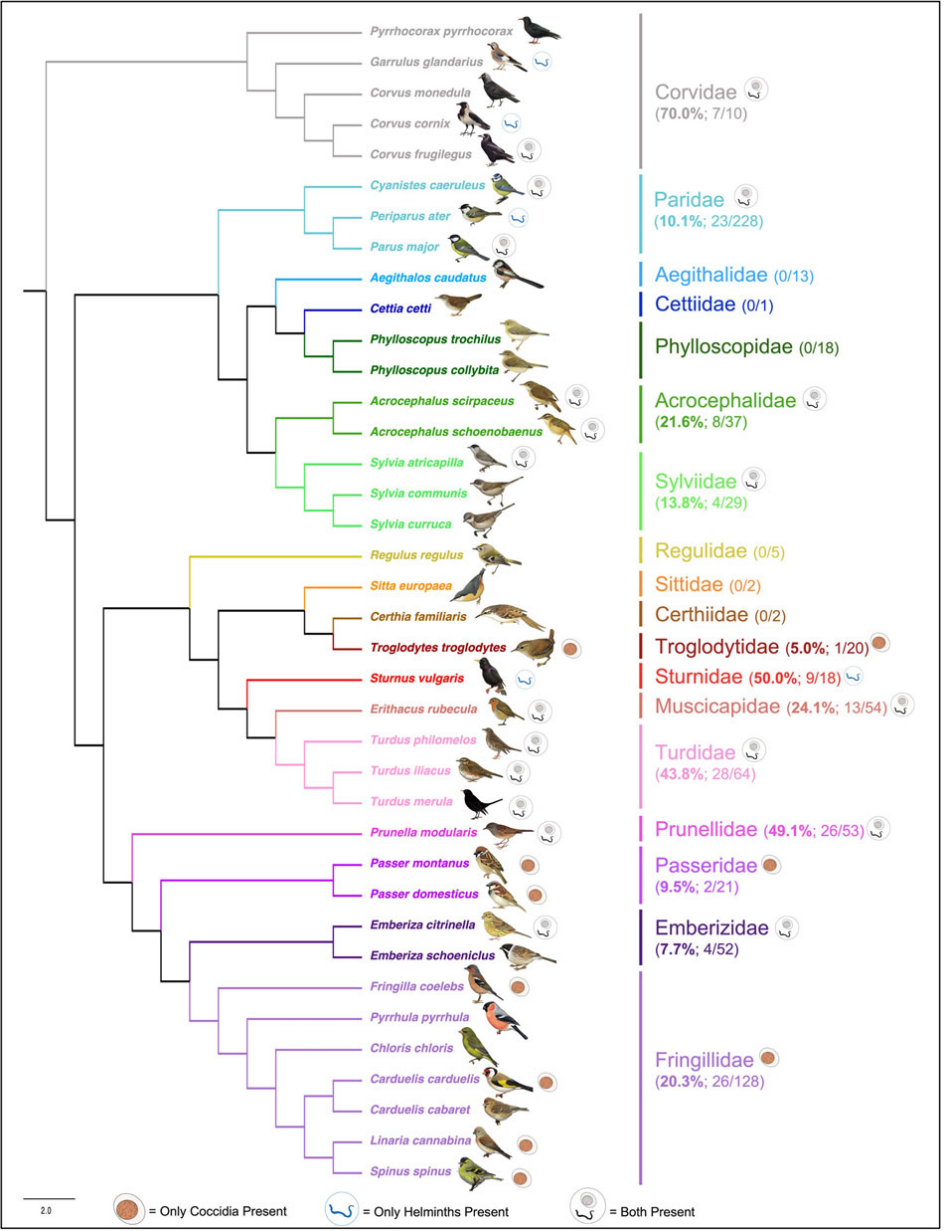
Cladogram of avian species sampled and their presence of parasites, generated from BirdTree (Jetz *et al*., 2012) and visualized in FigTree v1.4.4 (Rambaut, 2010). All bird images are from NatureGuides Ltd.

**Fig. 5.**
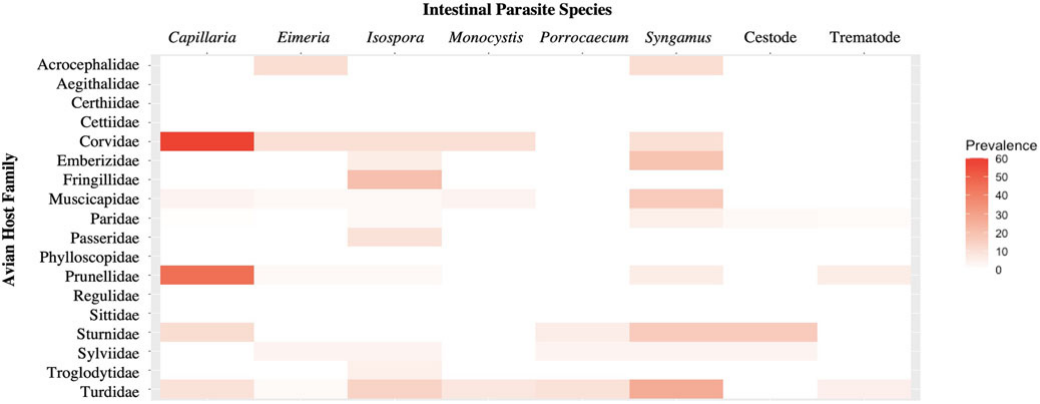
Heat map showing the prevalence rates (0–60%) of the various types of intestinal parasites in the avian host families sampled.

**Fig. 6.**
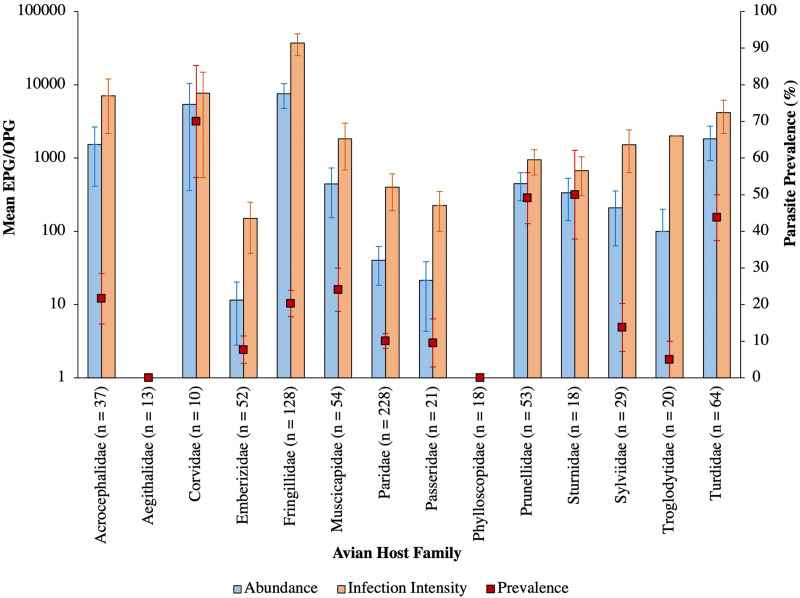
Mean abundance (±s.e.), infection intensity (±s.e.) and prevalence (±s.e.), represented by blue bars, orange bars and red square points, respectively, of intestinal parasites amongst avian families. Sample sizes are denoted in parentheses.

**Fig. 7.**
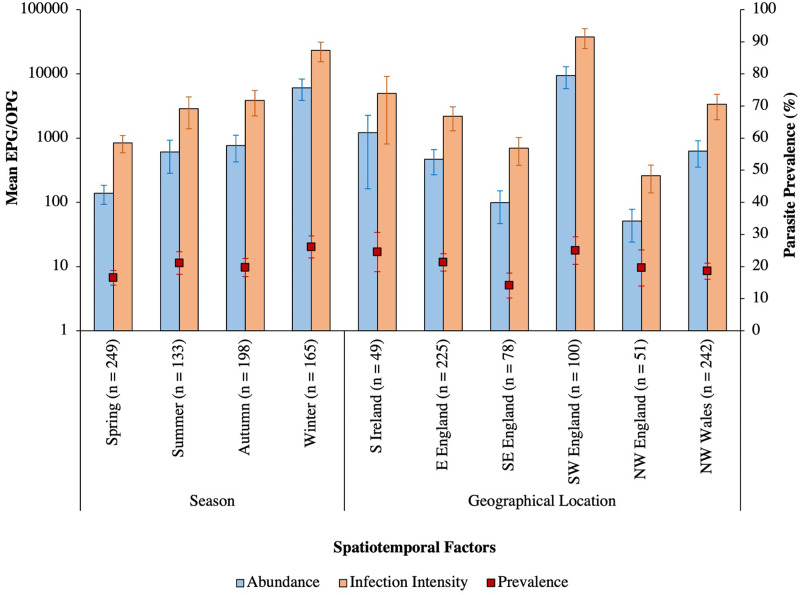
Mean abundance (±s.e.), infection intensity (±s.e.) and prevalence (s.e.), represented by blue bars, orange bars and red square points, respectively, of intestinal parasites amongst the sampled seasons and sites across the British Isles. Sample sizes are denoted in parentheses. N, north; E, east; W, west; S, south; EPG/OPG, eggs/oocysts per g.

Intestinal parasites were identified in 20.0% (151/755) of all birds sampled (Fig. 4). Phylogenetically controlled analysis revealed that the prevalence of intestinal parasites differed between avian host families and amongst seasons, but not between sites (Table 2; Figs 5–7). Overall prevalence differed between families (range: 0–70%; mean ± s.e.: 23.2 ± 5.6%) with 6 avian families being uninfected (Figs 4 and 5; Table 2). The highest prevalences were observed in corvids, starlings and dunnocks (Figs 5 and 6); meanwhile, long-tailed tits and leaf warblers had the lowest prevalences. However, rarefaction curves suggested that asymptotic richness has only been reached amongst Acrocephalidae, Paridae and Turdidae (Supplementary Fig. 2). *Syngamus* spp. eggs (33.1%; 50/151), *Isospora* spp. oocysts (32.4%; 49/151) and *Capillaria* spp. eggs (27.2%; 41/151) were the most detected genera (Fig. 5). Birds sampled in the winter months had the highest prevalence (26.1 ± 3.4%) while those sampled in spring had the lowest (16.5 ± 2.3%) (Fig. 7). Although there were no associations between parasite prevalence and feeder presence or habitat diversity (Supplementary Table 1), diet diversity and host age were strongly associated with prevalence (Table 2); in particular, infected birds had a greater mean diet diversity (2.7 ± 0.1) than those non-infected (2.6 ± 0.04), with species that were known to consume 6 diet types having the highest prevalence rates (75.0%; 6/8), followed by those with 4 (30.43%; 21/69), 2 (28.0%; 26/93), 1 (21.3%; 26/122) and 3 types (15.7%; 59/377). Juveniles had higher parasite prevalence of infection (22.4%; 89/397) than adults (17.9%; 51/285).

### Parasite abundance

**Fig. 8.**
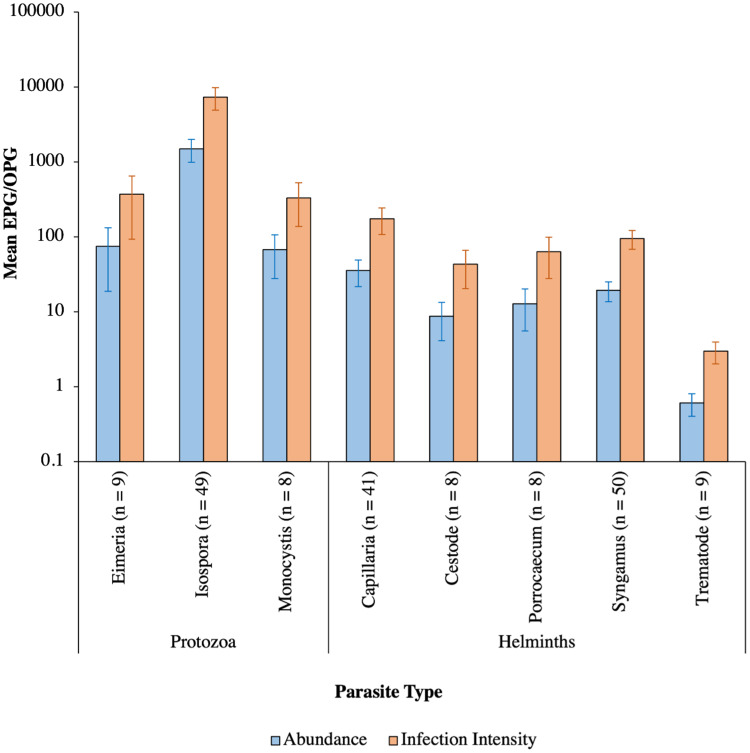
Mean abundance (±s.e.) and infection intensity (±s.e.) represented by blue and orange bars, respectively, of the various parasite genera in all or infected hosts. The number of individual hosts each parasite was detected in are denoted in parentheses. EPG/OPG, eggs/oocysts per g.

The mean abundance of parasitic EPG/OPG of feces across all birds was 1704.0 ± 504.8. *Isospora* had the highest abundance overall (1485.2 ± 500.51 OPG) and trematodes the lowest (0.6 ± 0.2 EPG**)** with parasite genus driving trends in abundance (Table 3; Fig. 8). Phylogenetically controlled analyses found that parasite abundance varied between avian host families (Table 1; Fig. 6); specifically, abundances were higher in thrushes (1826.6 ± 905.6 EPG/OPG) than in starlings (234.6 ± 181.5 EPG). None of geographical region, diet diversity or feeder presence appears to influence abundance, but season, habitat diversity and age were strongly influential (Table 1; Fig. 7); specifically, birds sampled in winter had the highest mean abundance (6080 ± 2197.4 EPG/OPG) and those sampled in spring had the lowest (139.0 ± 46.1 EPG/OPG). Birds that were known to occupy 3 habitat types had the highest abundance (3350.0 ± 1254.9 EPG/OPG) followed by those with 5 (1092.3 ± 780.0 EPG/OPG), 4 (987.3 ± 367.2 EPG/OPG), 2 (215.2 ± 91.5 EPG/OPG) and 1 type (3.1 ± 2.2 EPG/OPG). Juveniles were infected to a higher degree (2565.0 ± 915.9 EPG/OPG) than adults (841.6 ± 327.3 EPG/OPG).

**Table 3.** Significant results of phylogenetically controlled, Poisson MCMC GLMMs comparing the various avian parasite genera on abundance

		Estimate	95% CI		pMCMC
			Lower	Upper	
Intercept		−73.94	−110.52	−23.75	<0.001
Parasite genus					
*Capillaria vs*	Cestode	−10.37	−15.35	−5.39	<0.001
	*Eimeria*	−8.29	−12.90	−3.73	<0.001
	*Isospora*	4.65	1.39	8.31	0.006
	*Monocystis*	−8.92	−13.62	−4.33	<0.001
	*Porrocaecum*	−9.38	−14.03	−4.37	<0.001
	*Syngamus*	3.58	0.18	6.87	0.036
	Trematode	−9.00	−13.48	−4.39	<0.001
*Isospora vs*	Cestode	−10.29	−22.43	−4.84	<0.001
	*Eimeria*	−9.90	−21.07	−4.37	<0.001
	*Monocystis*	−10.50	−22.49	−5.27	0.040
	*Porrocaecum*	−10.92	−22.55	−5.10	<0.001
	*Syngamus*	1.68	−8.42	−5.95	<0.001
	Trematode	−11.08	−19.61	−6.14	<0.001
*Syngamus vs*	Cestode	14.81	3.23	20.24	0.017
	*Eimeria*	14.61	3.38	20.79	<0.001
	*Monocystis*	15.31	3.99	21.52	<0.001
	*Porrocaecum*	15.33	3.59	21.01	<0.001
	Trematode	−8.91	−20.46	−3.61	0.029
Random effects					
	Host phylogeny	9.43	0.003138	44.59	
	Time until analysis	16 946	0.002174	62.77	

Estimates, lower and upper 95% CI values, and *P* values (pMCMC) have been included for the intercept, random effects and each level of the factors.

### Parasite infection intensity

The mean infection intensity of parasitic eggs and oocysts within the infected birds was 8407.0 ± 2420.0/g of feces. *Isospora* had the highest maximum intensity overall (7327.5 ± 2417.7) and trematodes had the lowest maximum intensity (2.9 ± 0.9), with parasite genus driving trends in infection intensity (Table 4; Fig. 8). Phylogenetically controlled analyses found that infection intensity differed between avian host families (Table 5; Fig. 6); specifically, infection intensities were higher in finches (37 301.9 ± 12 331.8 OPG) than in accentors (908.8 ± 471.1 EPG/OPG). Although neither season nor diet diversity appeared to influence infection intensity, geographical region, habitat diversity and feeder presence were strongly influential (Table 5; Fig. 7); in particular, birds sampled in northwest England and southwest England had the lowest (260.0 ± 120.4 EPG/OPG) and highest (37 688.0 ± 12 822.8 EPG/OPG) average parasitic infection intensities, respectively (Fig. 7). Samples from areas with feeders had a greater infection intensity (9847.1 ± 2990.6 EPG/OPG) than those without (3894.1 ± 2370.9 EPG/OPG). Birds with 3 habitat types had the highest infection intensities (22 079.5 ± 7752.1 EPG/OPG), followed by those with 5 (4437.5 ± 3091.3), 4 (3184.7 ± 1142.0 EPG/OPG), 2 (1260.7 ± 451.7 EPG/OPG) and 1 habitat type (50.0 ± 0 EPG).

**Table 4.** Significant results of phylogenetically controlled, Poisson MCMC GLMMs comparing the various avian parasite genera on infection intensity

		Estimate	95% CI		pMCMC
			Lower	Upper	
Intercept		−9.34	−21.48	3.89	0.082
*Capillaria vs*	Cestode	−9.18	−13.57	−4.21	<0.001
	*Eimeria*	−8.89	−12.94	−4.41	<0.001
	*Monocystis*	−9.51	−13.86	−4.64	<0.001
	*Porrocaecum*	−9.77	−14.37	−5.41	<0.001
	*Trematode*	−9.30	−14.18	−5.14	<0.001
*Isospora vs*	Cestode	−11.98	−16.94	−7.74	<0.001
	*Eimeria*	−11.59	−15.57	−7.27	<0.001
	*Monocystis*	−12.19	−17.00	−8.17	<0.001
	*Porrocaecum*	−12.61	−17.06	−8.00	<0.001
	Trematode	−12.17	−16.47	−7.69	<0.001
*Syngamus vs*	Cestode	13.13	8.73	17.35	<0.001
	*Eimeria*	12.93	8.88	17.89	<0.001
	*Monocystis*	13.63	9.48	18.63	<0.001
	*Porrocaecum*	13.65	9.09	18.11	<0.001
	Trematode	−10.59	−14.96	−6.51	<0.001
Random effects					
	Host phylogeny	12.11	0.003134	60.18	
	Time until analysis	15 367	0.001573	59.87	

Estimates, lower and upper 95% CI values, and *P* values (pMCMC) have been included for the intercept, random effects and each level of the factors.

**Table 5.** Significant results of phylogenetically controlled, Poisson MCMC GLMMs comparing the various factors affecting avian parasitic infection intensity

		Estimate	95% CI		pMCMC
			Lower	Upper	
Intercept		6.86	−1.66	13.75	0.070
Avian family					
Fringillidae	Prunellidae	−2.40	−4.90	−0.19	0.048
Geographical region					
Northwest England	Southeast England	−0.39	−10.54	−10.46	0.034
	Southwest England	−0.51	−9.22	−8.03	0.041
Ecological variables					
	Habitat diversity	0.71	−1.33	−0.02	0.048
	Feeder presence (yes *vs* no)	−0.55	−2.50	−1.73	0.044
Random effects					
	Host phylogeny	1.52	0.002338	6.99	
	Time until analysis	15 937	0.002696	64.89	

Estimates, lower and upper 95% CI values, and *P* values (pMCMC) have been included for the intercept, random effects and each level of the factors.

### Rarefaction analyses

Rarefaction analyses on all samples suggest that we have detected over 80% of the parasite genera in Acrocephalidae, Muscicapidae, Paridae, Prunellidae and Turdidae (Supplementary Fig. 2). Analyses suggest we have detected 50% of parasite genera likely to be present in Corvidae, ~65% of parasite genera present in Emberizidae, ~50% of parasite genera present in Sturnidae and ~70% of parasite genera likely to be present in Sylviidae (Supplementary Fig. 2).

### Coinfections and co-occurrence analyses

**Fig. 9.**
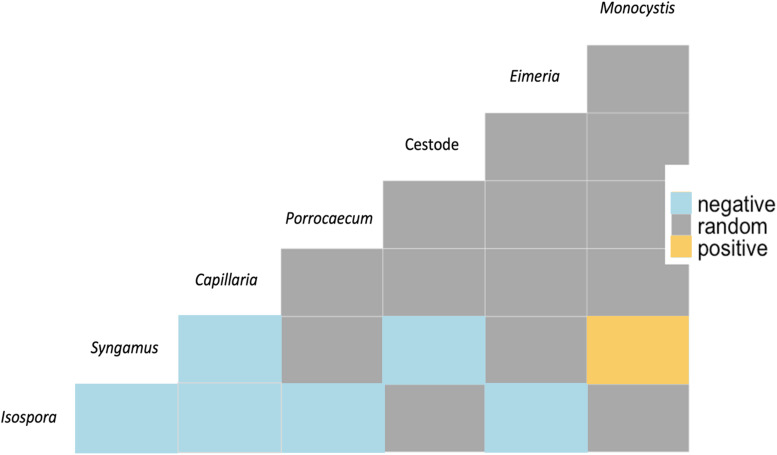
Co-occurrence matrix for intestinal parasite genera demonstrating non-random associations between helminths and protozoa in passerine samples. Yellow and blue denote positive and negative interactions, respectively, while grey represents random associations.

Most infected individuals had a solitary parasitic infection (87.4%; 132/151) while the remainder (12.6%; 19/151) had co-occurring infections; infection by 2 species was the most common (7.9%; 12/151), followed by 3 (2.0%; 3/151), 4 (2.0%; 3/151) and 5 (0.7%; 1/151). The most prevalent parasite genus, *Syngamus* spp., had the highest rates of co-occurring with *Capillaria* spp. in coinfections (Fig. 9). Co-occurrence analyses found both positive and negative associations between coinfecting parasite genera, with positive associations found only between *Monocystis* and *Syngamus*, and negative associations found amongst 6 pairs (Fig. 9). *Eimeria*, *Porrocaecum* and cestodes were only negatively associated with 1 other parasite while *Syngamus* and *Capillaria* had 2 negative correlations each and *Isospora* was negatively associated with 4 other parasites (Fig. 9).

## Discussion

Variation in the prevalence and abundance of intestinal parasites differed significantly between host families and between seasons; meanwhile, host family and geographical region appeared to affect parasitic infection intensity. Trends in parasite abundance and infection intensity were driven by parasite genus, specifically *Capillaria*, *Isospora* and *Syngamus*, although habitat diversity and host age also influenced abundance, and infection intensity was more than double at sites with feeders. Parasite prevalence was strongly affected by diet diversity and host age.

Previous epidemiological studies examining the distribution and intensity of endoparasitic infections in avian hosts have been carried out using a wide range of taxonomic groups, including Galliformes, Passeriformes and Psittaciformes (e.g. Masello *et al*., 2006; Pérez Cordón *et al*., 2009); although these have been performed in a wide range of countries, Britain and Ireland have not been thoroughly examined. Passerines can be parasitized by a diverse array of generalist and specialist protozoan and helminthic organisms, with varying pathogenicities (Brown *et al*., 2010; Schoener *et al*., 2012, 2013); the overall infection rate of 20% reported here is lower than those previously detected at 51.6, 34.8 and 29–48% from a range of birds in Spain, Iran and Bangladesh, respectively (Badparva *et al*., 2014; Hoque *et al*., 2014). However, these studies used larger amounts of feces and a wider range of diagnostic methods, and studied mainly domestic, as opposed to wild, birds, which may explain the lower prevalence rates we observed (Badparva *et al*., 2014; Hoque *et al*., 2014). Indeed, our findings are comparable to the prevalence of 20% detected in resident wild birds in Bangladesh (Hoque *et al*., 2014). This variation of intestinal parasites amongst species could be due to coevolutionary adaptations caused by geographical and climatic variation or host immunological status and genetic resistance. Coevolution can be seen in birds that have adapted to be more tolerant to certain parasites and are known as natural hosts (Granthon and Williams, 2017); meanwhile, immune status may be affected by stressors, such as breeding, competition, nutrition and age (Hudson, 1986; Zuk *et al*., 1990; Møller, 1991; Isomursu *et al*., 2006; Bandelj *et al*., 2015). In this study, juveniles had greater parasite prevalence and abundance rates than adults; this is different to prior studies on haemoparasitic infections in birds over time, with Piersma and van der Velde (2012) reporting malaria in 77% of adults but none in fledglings (Sanz *et al*., 2001; Atkinson and Samuel, 2010). The variations seen here may be due to the immunological naïvety of the host, with older birds developing robust responses to previously encountered infections (Isomursu *et al*., 2006; Sorci, 2013; Bandelj *et al*., 2015).

Spatiotemporal variation in endoparasitic infections have been reported from biogeographic and evolutionary studies exploring the prevalence and abundance of intestinal parasites (Santiago-Alarcon *et al*., 2019; Bodawatta *et al*., 2020); such studies explore the changes in parasite communities across defined time periods and spatial regions and can be essential in elucidating the shedding patterns of eggs and oocysts, such as the fecal excretion of *Isospora* spp. on a diurnal circadian cycle (Martinaud *et al*., 2009; Dolnik *et al*., 2011). The seasonal variation observed in the current study may be due to temperature, humidity or precipitation; these have all been proven to affect the viability of parasites and, particularly, their infective stages when in the environment (Langkjær and Roepstorff, 2008; Maya *et al*., 2012). The findings of the highest parasite prevalence in winter, often due to isosporiasis, and the lowest in spring, may have been due to existing infections that may persist in winter as birds may not have many resources to invest in immunity over this period or be due to the increased sociality of birds forming groups in winter (Nord *et al*., 2020). Meanwhile, birds sampled in spring may have had reduced prevalence and abundance of parasites due to decreased rainfall or moisture content in the air; eggs and oocysts can desiccate faster under dryer conditions, as seen in ascarid helminths (Senecal *et al*., 2020), but recording of specific weather conditions would be required to test this.

The geographical variation may be due to the type of habitat the birds reside in as well as the availability of food, which may be affected by anthropogenic activities (Aponte *et al*., 2014). The ubiquity of wild birds allows them to inhabit an array of habitats, from urban to rural landscapes (Bairlein, 1983). Although the passerine species in this study were sampled in farmland, woodland or garden sites, the diversity of their habitat use varies (Storchová and Hořák, 2018). Host habitat diversity, in particular, was found to have a strong influence on parasite abundance and infection intensity, with observed overall trends of greater abundance and infection intensity rates as mean habitat diversity increased, aside from the several *Isospora*-infected finches leading to the abnormally high rates seen in birds that occupy 3 habitat types. These findings may also have contributed into the overall significance of geographical variation in avian parasite epidemiology; for example, prior research has confirmed the importance of ecology in spatial distribution of haemoparasites (Cornuault *et al*., 2013). Meanwhile, food availability is known to affect avian life-history traits, such as reproductive success and body mass (Récapet *et al*., 2017). The effect of humans on wildlife is multifaceted due to urbanization and can include outwardly positive actions, such as the widespread use of bird feeders to provide supplementary food to wild animals and which appear to have been responsible for some variation in infection intensity (Delgado-V and French, 2012; Becker *et al*., 2018); however, they can act as a source of parasite transmission, such as in trichomoniasis (Lennon *et al*., 2013).

Variation in diet can lead to certain birds having a greater likelihood of ingesting potential intermediate hosts, such as parasitized earthworms (Aponte *et al*., 2014), as evidenced by the potential case of pseudoparasitism by *Monocystis*, an earthworm parasite, we detected. Diet has the potential to influence the immune system by creating a stronger response to infection following the consumption of foods with antiparasitic activity; a variety of animals ingest items due to the therapeutic action of the secondary metabolites, such as parrots (Masello *et al*., 2018). The diverse feeding behaviours of passerines, which is dependent on their diet and includes ground foraging, can also contribute to parasite transmission (Willson, 1974), and further work should examine the potential relationship between diet and parasitic infection. For example, birds such as corvids, that consume anthropogenic foods may have fewer parasites; ring-billed gulls (*Larus delawarensis*) were found to have fewer trophically transmitted protozoa and helminths when their diet was formed primarily by these foods rather than aquatic intermediate hosts, such as fish or invertebrates (Aponte *et al*., 2014). Some diets might provide birds with more resources or energy that can be placed into mounting an immune response, or vice versa if the food is of poor quality (Strandin *et al*., 2018), and diet choice can also contribute to parasite transmission (Willson, 1974), which may explain the overall trend of greater parasite prevalence rates with increasing mean diet diversity seen here. Birds, such as most thrushes (Turdidae) and dunnocks (*Prunella modularis*), have a higher exposure to trophically transmitted eggs and oocysts through the wider range of foods they eat, which may explain the high incidences detected here (Dolnik *et al*., 2010); in the Netherlands, omnivorous passerines such as starlings (Sturnidae) were more heavily parasitized by helminths than seed eaters, such as finches (Fringillidae) (Borgsteede *et al*., 2000). Our finding of higher infection intensities in birds at sites with feeders highlights the potential risks of supplementary feeding for parasite transmission (e.g. Lennon *et al*., 2013).

Co-occurrence analyses demonstrated a positive association between *Monocystis* and *Syngamus* spp., which may be explained by earthworms serving as a definitive host and paratenic vector to both, respectively (Clapham, 1934; Field *et al*., 2003). Negative associations between *Isospora* and the 4 helminthic genera may be due to the varying immunological responses that occur within a host, particularly if they are bottom-up controls or resource-based competition of host nutrients from blood (Graham, 2008); meanwhile, *Isospora* and *Eimeria* spp. could be negatively associated due to their high relatedness leading to heterologous, or cross-protective, immunity (Smith *et al*., 2001). Moreover, host-mediated responses could explain the opposing correlation between *Syngamus* and *Capillaria* spp. or cestodes, as often infections with 1 type of helminth have led to protection against others (Cox, 2001); despite the first 2 species often being present in samples together, this negative association could be indicative of opposing abundances due to the clearing of secondary infections. Despite finding multiple associations between co-occurring parasites, we can only speculate as to whether these are due to genuine interactions.

While these potential mechanisms may underlie our observations, our rarefaction analyses suggest that we may have detected most intestinal parasite fauna in only 5 bird families, so further sampling to confirm parasite species richness is required. Additionally, we only used morphological analysis to identify species, using a single detection technique. The detection of trematode and eimeriid species could have been improved through sedimentation with salt or sugar solutions and sporulation, respectively, and may explain our low rates of detection of these taxa (Lobos-Ovalle *et al*., 2021; Abdu *et al*., 2022). More sensitive identification techniques, such as polymerase chain reaction followed by sequencing, used alongside morphological analyses, would allow the identification of specific parasite strains to confirm whether cross-species parasite transmission may be occurring (Nadler and De León, 2011; Perkins *et al*., 2011). Sampling throughout the day may increase the likelihood of detecting parasites in the prepatent stage; for example, egg excretion of helminths has been seen to occur during certain patterns in a day in natural or experimental avian infections (Villanúa *et al*., 2006; Wongrak *et al*., 2015). Thus, future studies should explore the epidemiology of these organisms in more controlled settings, such as regular sampling in the same site at a similar time every day.

Epidemiological studies are essential to further the understanding required for species conservation as well as research into host–parasite biodiversity, evolution and the transmission of parasitic disease to other organisms. Although most avian endoparasites can be non-pathogenic for healthy, adapted hosts, transmission to naïve bird species can decimate populations; for instance, non-adapted little penguins (*Eudyptula minor*) infected by avian malaria introduced by other hosts in New Zealand (Cannell *et al*., 2013; Sijbranda *et al*., 2017). Anthropogenic activities, such as habitat fragmentation through urbanization and climate change causing vector migration, can lead to increased stress on passerine hosts, which can decrease their immunological status to a level where these infections become fatal (Laurance *et al*., 2013). These studies can be helpful to the One Health approach currently gaining traction worldwide, due to the global distribution of passerines and increasing incidence of infectious and/or zoonotic diseases; however, the intrinsic value of the opportunistic nature of this kind of epidemiological research is in understanding the parasite faunas shared by disparate avian hosts from a variety of locations over time.

## Data Availability

Data will be available on open access upon acceptance of manuscript.

## Supplementary material

For supplementary material accompanying this paper visit https://doi.org/10.1017/S0031182022001779. click here to view supplementary material
