# Supplementary material for: Epidemiology of protozoan and helminthic parasites in wild passerine birds of Britain and Ireland
Source: Parasitology. 2023 Jan 4;150(3):297–310. doi: 10.1017/S0031182022001779 (PMC10090598; doi:10.1017/S0031182022001779)
Supplement: Supplementary file 1 [file S0031182022001779sup001.zip › S0031182022001779sup005.pdf]

Supplementary Table 2: Faecal dropping sizes for the various bird species sampled.

**Avian Species**	**Range (g)**		**Median (g)**
	**Minimum**	**Maximum**	
Acrocephalus schoenobaenus	0.01	0.14	0.05
Acrocephalus scirpaceus	0.01	0.19	0.04
Certhia familiaris	0.03	0.06	0.05
Cettia cetti	0.03	0.03	0.03
Corvus cornix	0.04	0.06	0.05
Corvus frugilegus	0.32	1.6	1.09
Corvus monedula	0.04	0.04	0.04
Garrulus glandarius	0.01	0.04	0.03
Pyrrhocorax pyrrhocorax	0.47	0.47	0.47
Prunella modularis	0.01	0.21	0.07
Emberiza citrinella	0.01	0.29	0.07
Emberiza schoeniclus	0.01	0.18	0.04
Acanthis cabaret	0.05	0.05	0.05
Carduelis carduelis	0.01	0.12	0.03
Chloris chloris	0.01	0.16	0.07
Fringilla coelebs	0.01	0.23	0.05
Linaria cannabina	0.02	0.14	0.06
Pyrrhula pyrrhula	0.03	0.24	0.19
Spinus spinus	0.01	0.09	0.03
Passer domesticus	0.01	0.08	0.04
Passer montanus	0.01	0.05	0.03
Phylloscopus collybita	0.01	0.05	0.03
Phylloscopus trochilus	0.01	0.05	0.03
Regulus regulus	0.01	0.02	0.02
Erithacus rubecula	0.01	0.21	0.05
Sitta europaea	0.05	0.06	0.06
Sturnus vulgaris	0.01	0.39	0.09
Sylvia atricapilla	0.01	0.5	0.14
Sylvia communis	0.01	0.23	0.06
Sylvia curruca	0.05	0.05	0.05
Aegithalos caudatus	0.01	0.07	0.03
Cyanistes caeruleus	0.01	0.18	0.05
Parus major	0.01	0.16	0.05
Periparus ater	0.01	0.1	0.04
Troglodytes troglodytes	0.01	0.07	0.03
Turdus iliacus	0.01	0.84	0.24
Turdus merula	0.01	0.55	0.15
Turdus philomelos	0.01	0.35	0.13
