# Supplementary figures and images for: Epidemiology of protozoan and helminthic parasites in wild passerine birds of Britain and Ireland
Source: Parasitology. 2023 Jan 4;150(3):297–310. doi: 10.1017/S0031182022001779 (PMC10090598; doi:10.1017/S0031182022001779)

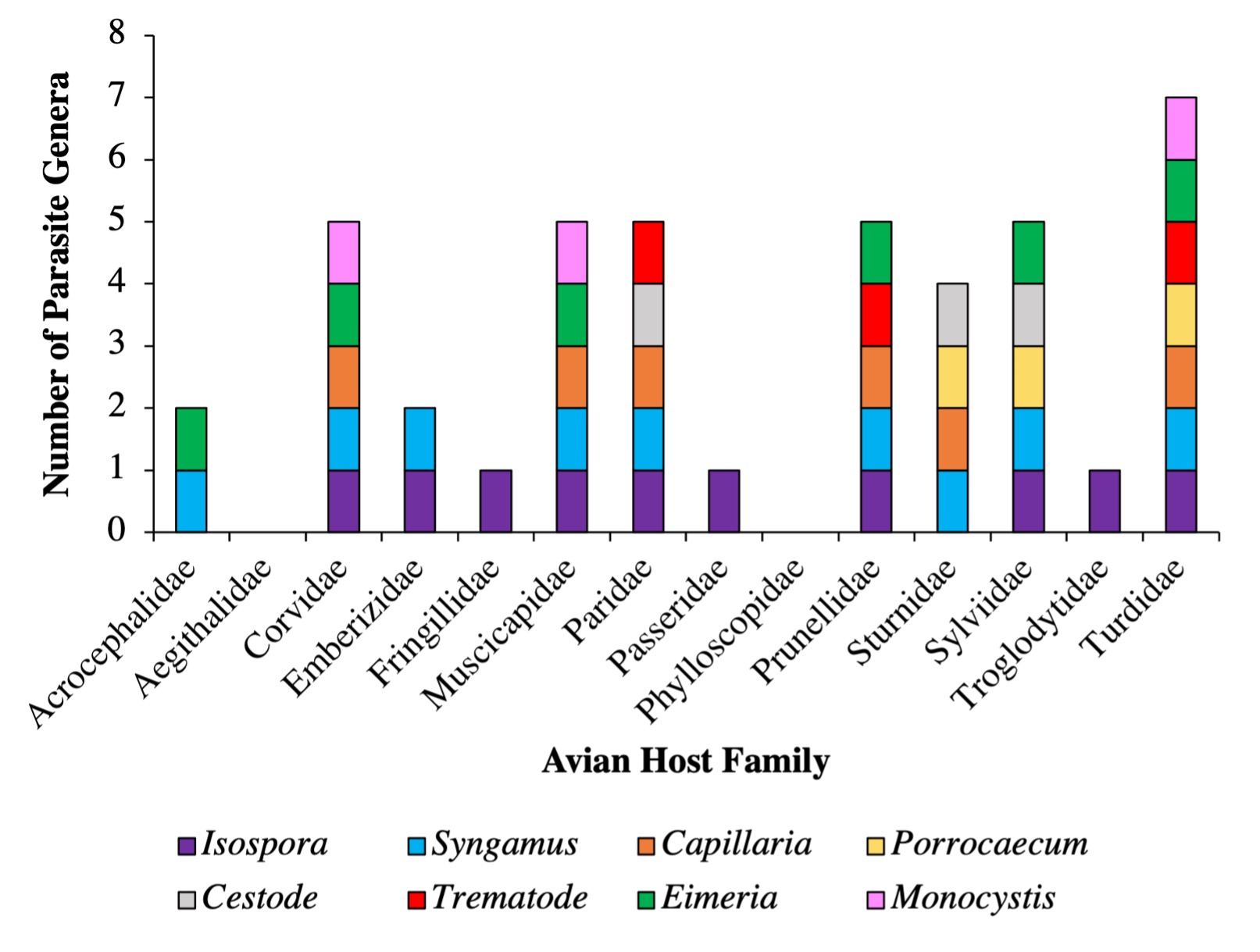

Supplement: Supplementary file 1 [file S0031182022001779sup001.zip › S0031182022001779sup002.tif]

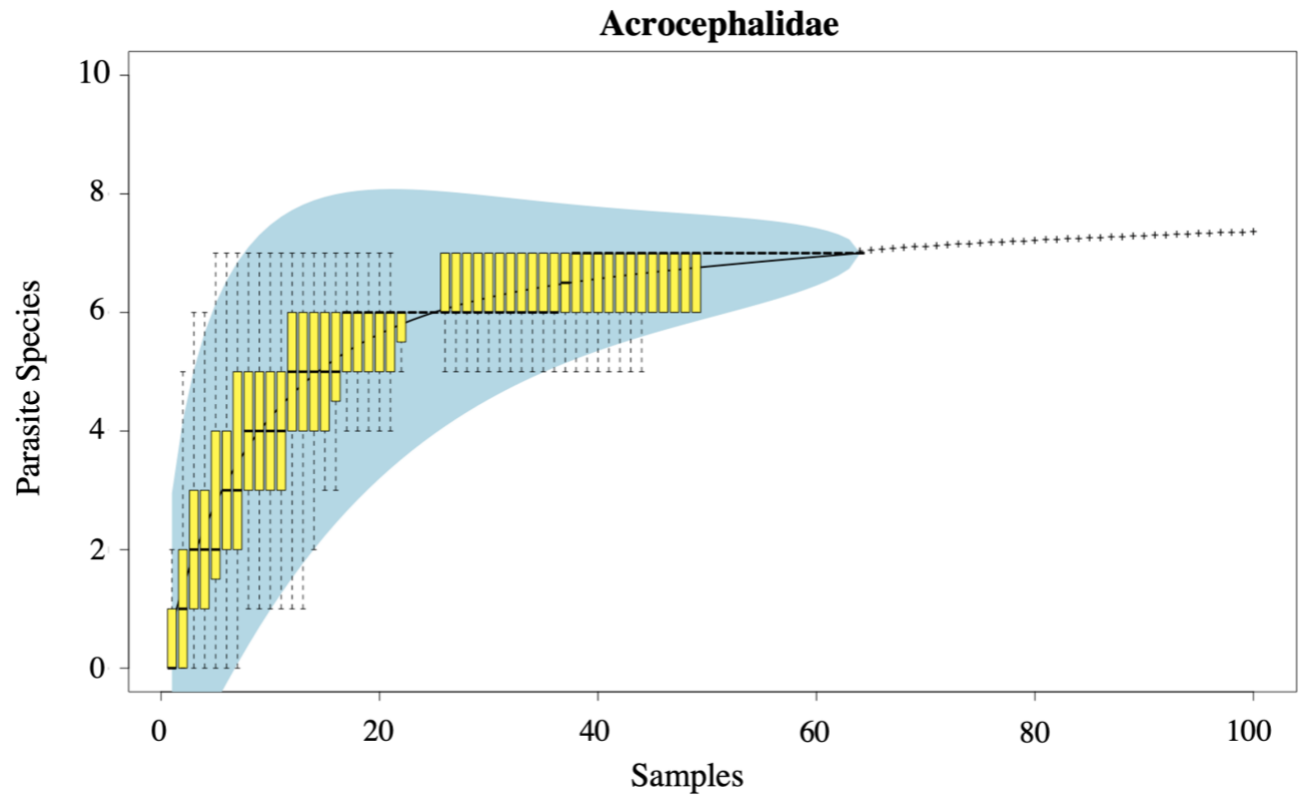

a)

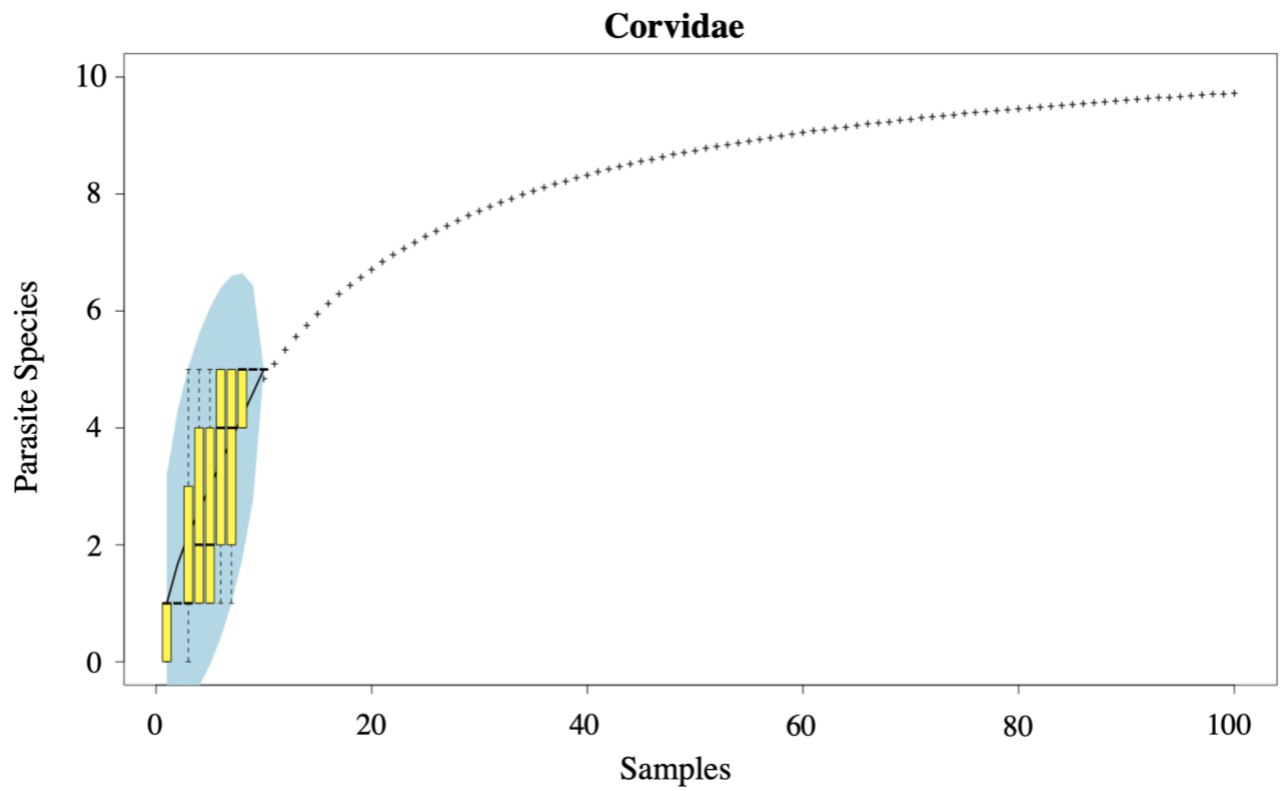

b)

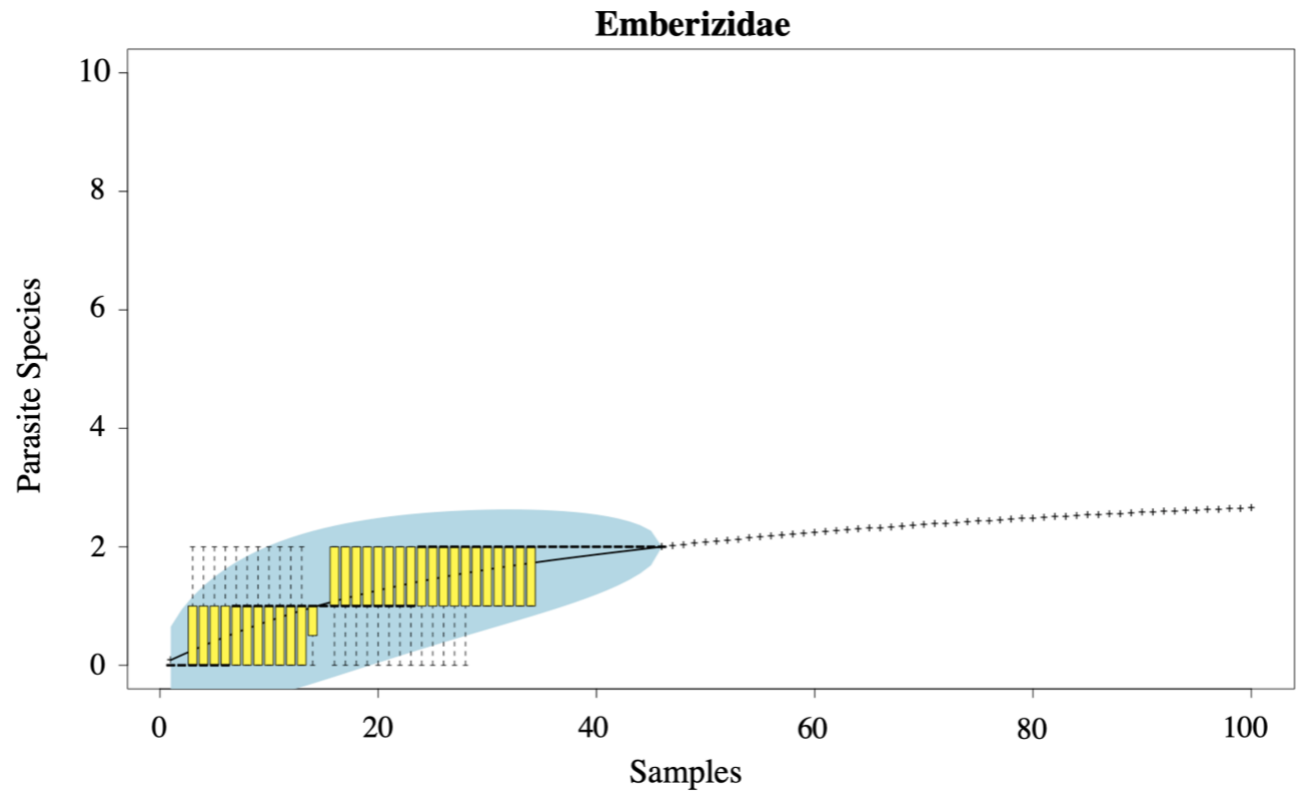

c)

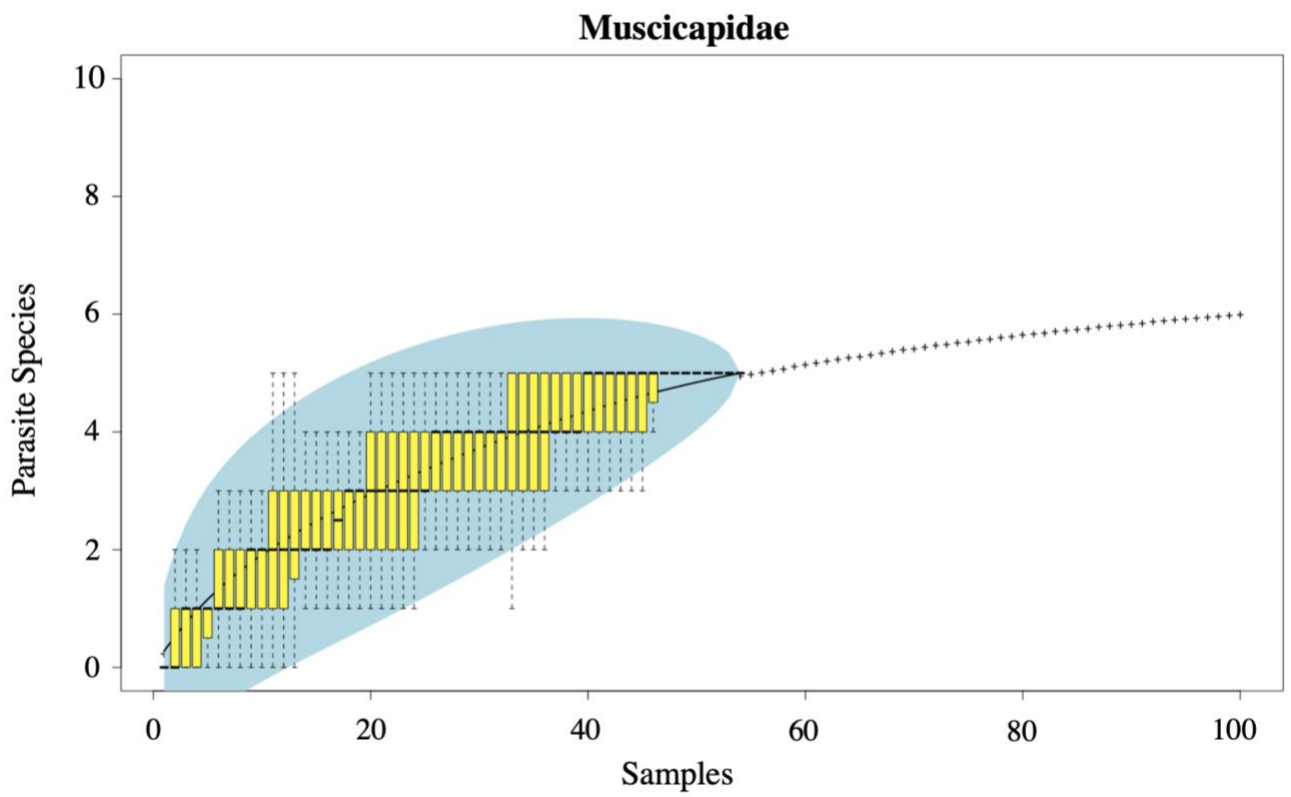

d)

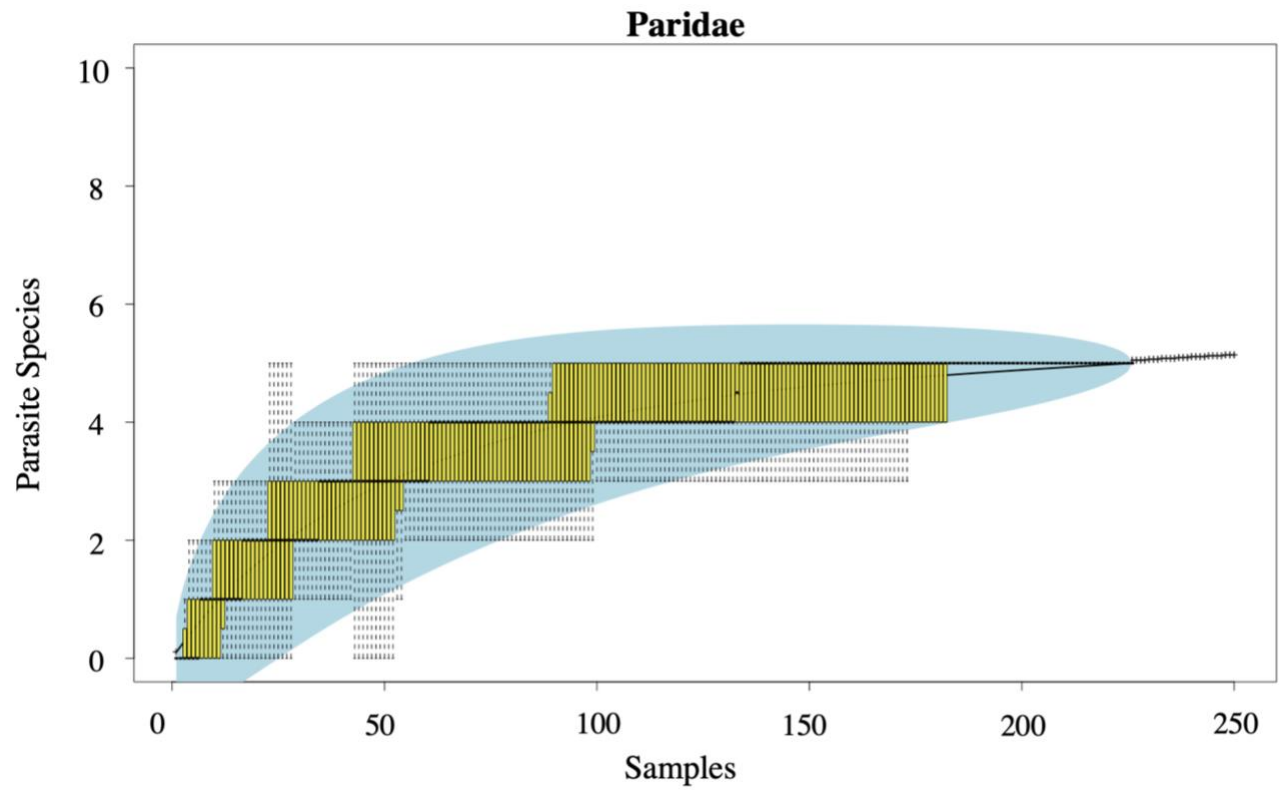

e)

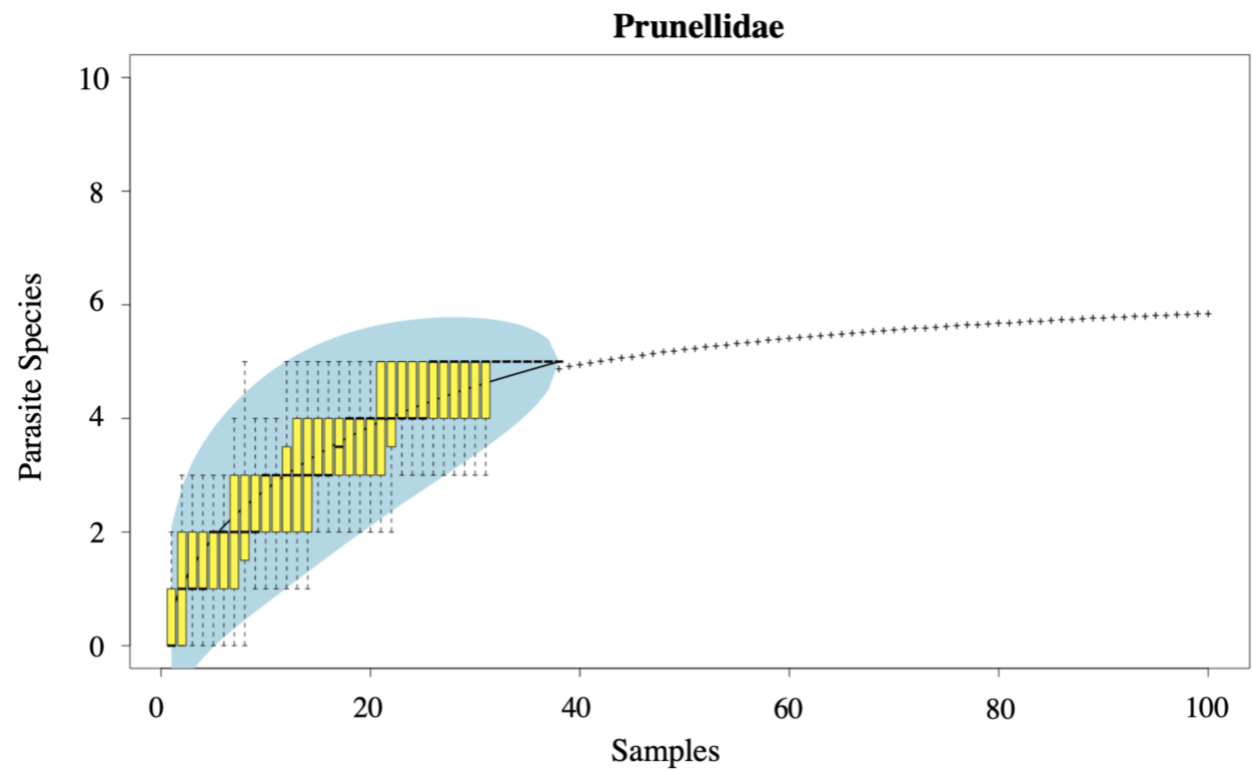

f)

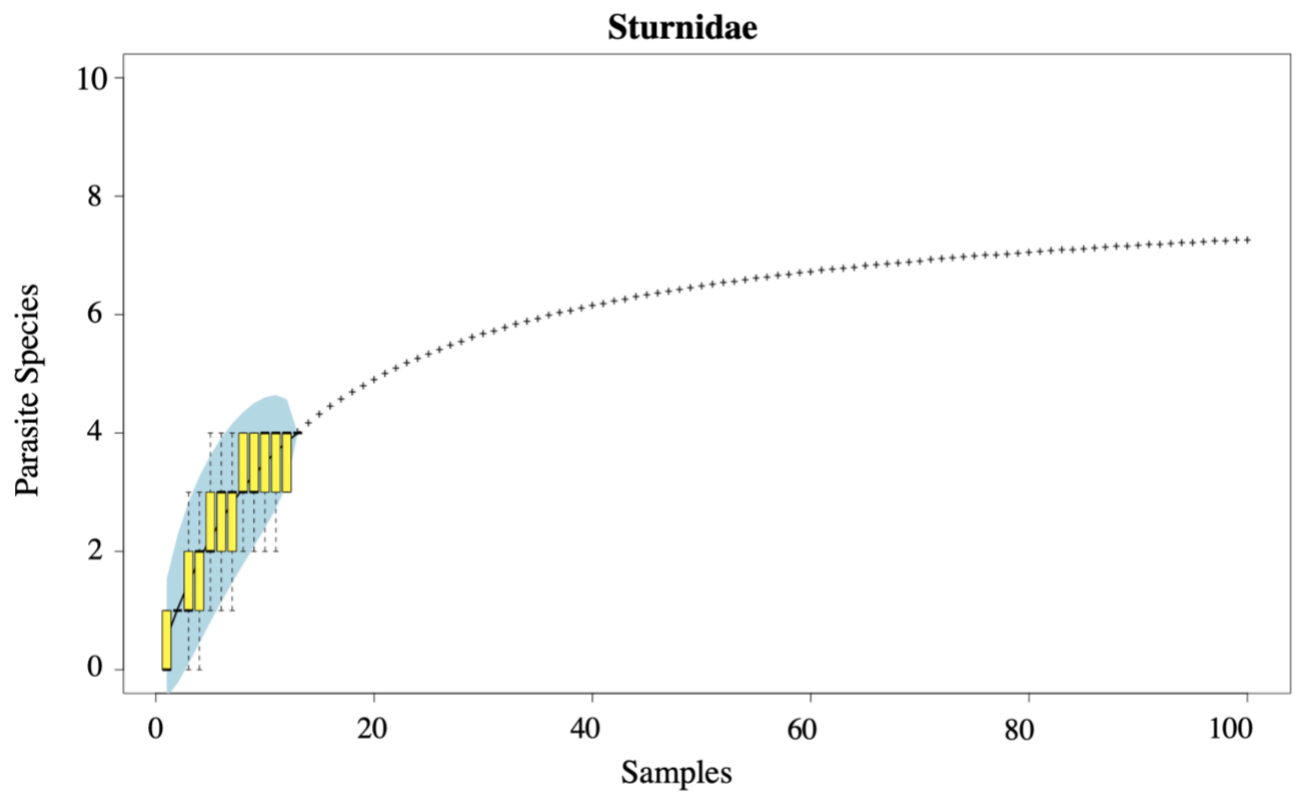

g)

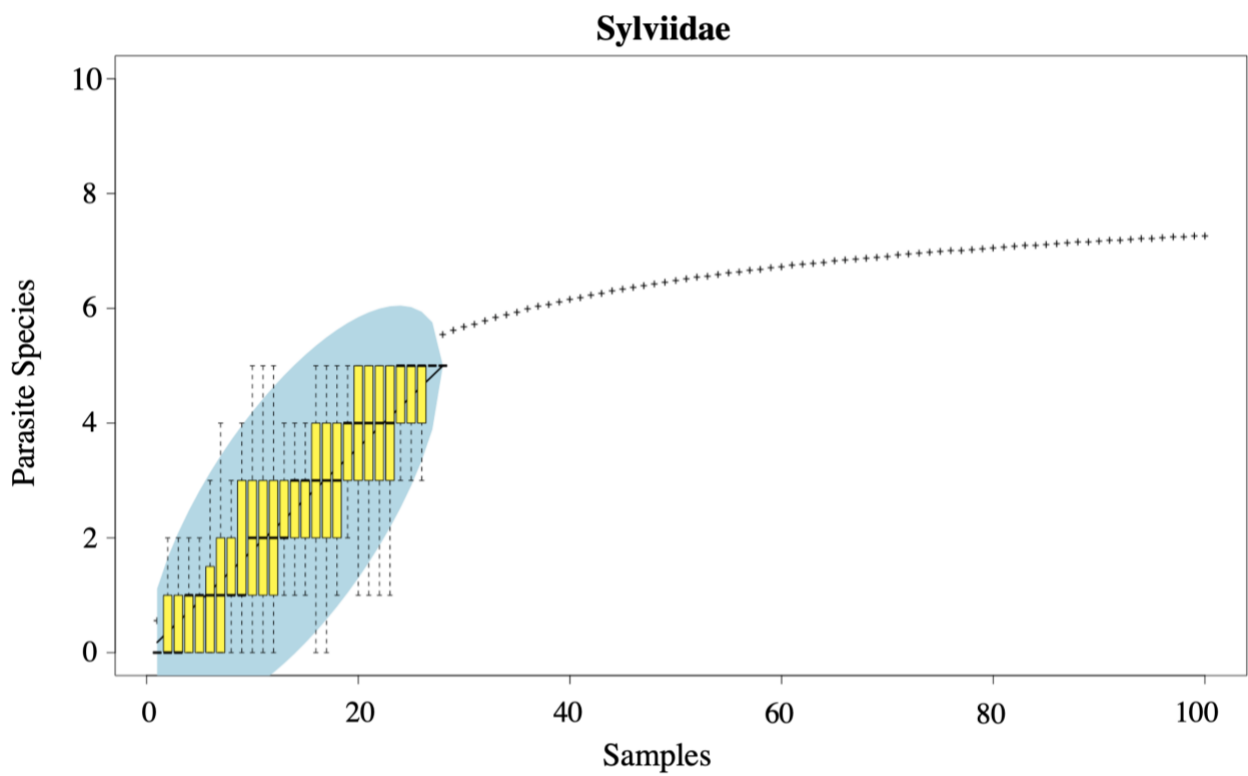

h)

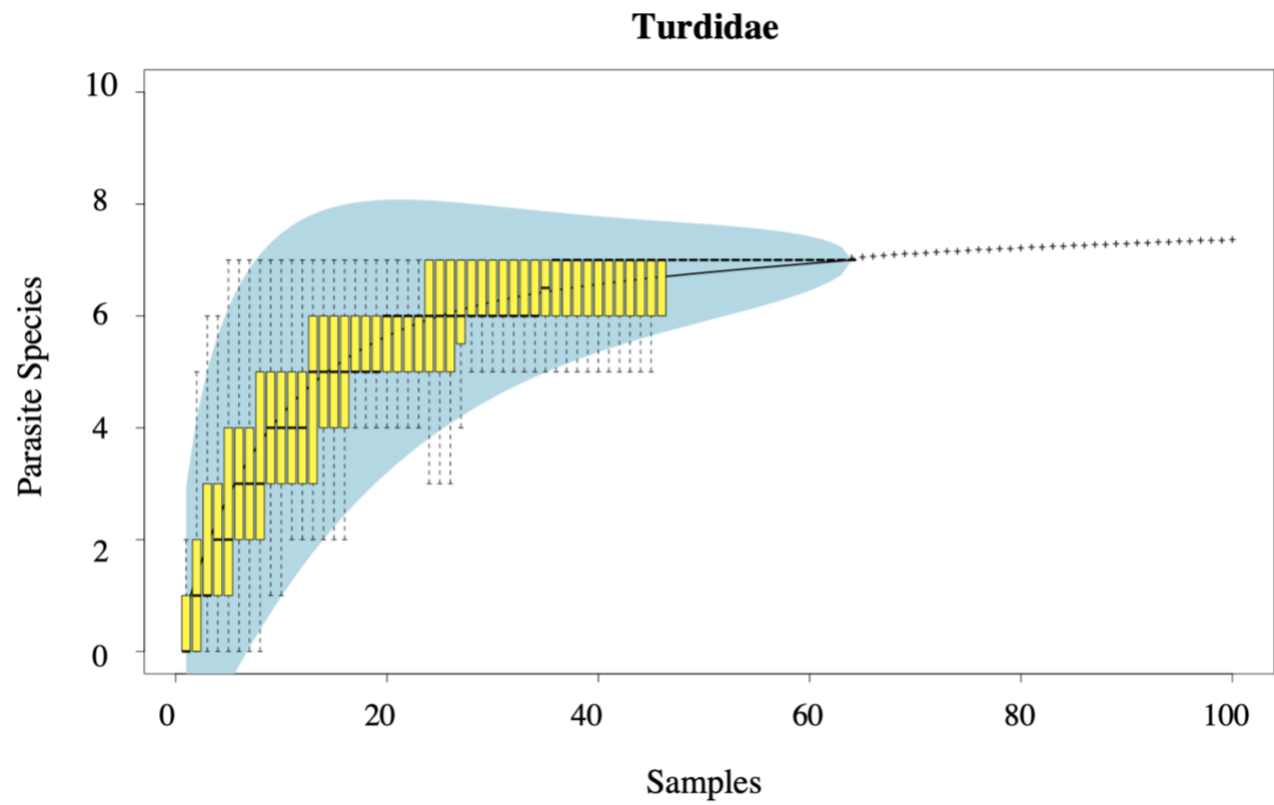

i)

Supplement: Supplementary file 1 [file S0031182022001779sup001.zip › S0031182022001779sup003.pdf]
